# Evaluation of immune suppressive mechanisms in a murine model of familial hemophagocytic lymphohistiocytosis

**DOI:** 10.1186/1546-0096-9-S1-P309

**Published:** 2011-09-14

**Authors:** Stéphanie Humblet-Baron, Adrian Liston

## Background

Familial hemophagocytic lymphohistiocyosis is a severe inflammatory condition due to genetic defect in cytotoxic activity (e.a. :perforin). This disease is similar to the macrophage activated syndrome (MAS).

In this disorder cytotoxic CD8 lymphocytes are highly proliferating due in part to a cytokine storm condition (IFNγ mediated). In this inflammatory climate macrophages gets activated and are forming fused cells called histiocytes which are pathognomonic for the disease.

## Aim

Using murine model, we are interested in studying the immune suppressive mechanisms of this disease to understand the reason why they fail to prevent the inflammation onset.

## Results

As previously shown, perforin deficient mice do not have significant immunophenotype difference compared to wild type (wt) controls (percentage and absolute number of CD8, CD4, B cells and NK cells).

In the regulatory T cell compartment (Treg) which are specifically natural immunosuppressive cells, the percentage of Treg between perforin deficient mice and wt are similar from the thymic formation to the periphery (percentage of Treg from the total splenic lymphocytes: wt: 2,7% (mean) +/- 0,8333 (SD), perforin deficient: 2,228% +/- 0,6372). Moreover their homing phenotype does not differ from wt for molecules such as CCR6, CD103, CTLA-4, CCR4, CCR7, CD62L.

In vitro supressive assay based with CFSE labeling showed a normal suppressive function for perforin deficient Treg to suppress wt effector T cells.

For the forthcoming meeting, comparison of the rescue ability of the wt and perforin deficient Treg in an autoinflammatory model in vivo (Treg depleted mice) will be presented.

